# Epidemiological and clinical characteristics of childhood pandemic 2009 H1N1 virus infection: an observational cohort study

**DOI:** 10.1186/1471-2334-11-225

**Published:** 2011-08-24

**Authors:** Jung-Woo Rhim, Kyung-Yil Lee, You-Sook Youn, Jin-Han Kang, Ji-Chang Kim

**Affiliations:** 1 Departments of Pediatrics, College of Medicine, The Catholic University of Korea, 505 Banpo-dong, Seocho-gu, Seoul 137-701, Republic of Korea; 2 Department of Pediatrics, The Catholic University of Korea, Daejeon St. Mary's Hospital, 520 Daeheung-dong, Jung-gu, Daejeon 301-723, Republic of Korea; 3 Department of Radiology, The Catholic University of Korea, Daejeon St. Mary's Hospital, 520 Daeheung-dong, Jung-gu, Daejeon 301-723, Republic of Korea

## Abstract

**Background:**

There was a pandemic influenza around the world in 2009 including South Korea since last pandemic occurred four decades ago. We aimed to evaluate the epidemiological and clinical characteristics of this infection in childhood.

**Methods:**

We evaluated the epidemiologic characteristics of all the subjects infected with the 2009 H1N1 influenza A virus (2,971 patients, ≤ 15 years of age), and the clinical and laboratory findings of the inpatients (217 patients, 80 had pneumonia) between 1 September 2009 and 31 January 2010 in a single hospital throughout the epidemic.

**Results:**

The age distribution of all the subjects was relatively even. Over 90% of cases occurred during a two-month period. Two hundred and five patients (94.5%) received oseltamivir within 48 h of fever onset, and 97% of inpatients defervesced within 48 h of medication. The group with pneumonia included more males than females, and had higher leukocytes counts with lower lymphocyte differentials than the group without pneumonia. The white blood cell count and lymphocyte differential were associated with the severity of pneumonia. Corticosteroid treatment for severe pneumonia patients was highly effective in preventing disease progression.

**Conclusion:**

Children of all ages affected with even rates of infection, but males were predominant in pneumonia patients. Pneumonia patients showed lymphopenia and its severity was associated with the severity of illness. Our results suggest that the mechanism of lung injury in 2009 H1N1 virus infection may be associated with the host immune response.

## Background

Although influenza virus infection has been a major global concern since the pandemic 1918 'Spanish flu', there have been no pandemic influenzas for near four decades after the 1968 'Hong Kong flu'. The pandemic 2009 H1N1 influenza A (2009 H1N1) virus infection was reported first in Mexico in February 2009, and then the virus spread rapidly worldwide, including in South Korea [1]. It has been reported that the majority of H1N1 patients were children and young adults and the mortality rate was not higher than that of seasonal influenza [2-4]. The majority of patients affected by the 2009 H1N1 virus infection recovered uneventfully, but some previously healthy patients developed a rapidly progressive pneumonia, leading to acute respiratory distress syndrome (ARDS), multi-organ failure, and death [5-7]. With this enigma, the pathogenesis of acute lung injury (pneumonia) in influenza infections remains unknown [1,8,9].

In South Korea, the first patient with 2009 H1N1 virus infection was reported in May, 2009. The number of patients gradually increased until mid-October, when the number of patients was overwhelming for a month, and then gradually decreased, with few people becoming infected after February, 2010. Owing to sensational reports of childhood fatality in the mass media and a new diagnostic tool, the real-time reverse transcriptase-polymerase chain reaction (RT-PCR), we had the opportunity to evaluate patients with 2009 H1N1 virus infection from the onset of their illness. In addition, during the study period we observed a dramatic effect of early treatment with corticosteroids and oseltamivir for patients with severe pneumonia including rapidly progressive pneumonia [9,10]. In this study, we evaluated the epidemiological, clinical and laboratory features of children with 2009 H1N1 virus infections in a single hospital throughout the epidemic.

## Methods

We retrospectively evaluated all patients with 2009 H1N1 virus infection during the pandemic (2,971 patients) for epidemiologic characteristics, and for clinical characteristics, we reviewed the medical records and chest radiographic findings of 217 children admitted to The Catholic University of Korea, Daejeon St Mary's Hospital between 1 September 2009 and 31 January 2010. The diagnosis of patients depended on positive results for the 2009 H1N1 virus RT-PCR (AccuPower^™ ^in Korea, BiONEER, Alameda, CA, USA) through throat swabs.

Although indications for admission were not clearly defined in this study, the majority of the inpatients were those who were suspected to have severe disease such as pneumonia and to have risk factors for severe disease such as infants and bronchial asthma. However, it might be possible that excessive concern of parents on fatality of this infection in part affected on admission of the uncomplicated cases. Among the 217 inpatients, we selected 80 patients with pneumonia and 137 patients without pneumonia, based on the chest radiographs. The chest radiographic patterns of admitted patients were reviewed and classified by two pediatricians (KY Lee and JW Rhim) and one pediatric radiologist (JC Kim). The patients with chest radiographic patterns that showed increased nodular densities along the bronchial trees unilaterally or bilaterally, were designated the bronchopneumonia group (49 patients). Patients with a distinctive large patch of infiltration, segmental or lobar consolidations were designated the segmental/lobar pneumonia group and regarded as having a severe pneumonia (31 patients). The first day of fever was regarded as the first day of illness. Among pneumonia patients, we tried corticosteroid treatment for 17 patients with severe pneumonia. As for indication of corticosteroids (methyprednisolone, MP or prednisolone), the subjects had severe respiratory distress with hypoxemia at presentation (12 cases) or during hospitalization (5 cases) requiring O_2 _therapy (9,10). We compared the clinical and laboratory characteristics of the different groups.

The study was approved by the Institutional Review Board of the Catholic University of Korea, Daejeon St Mary's Hospital.

### Statistical analysis

Statistical analyses were performed using the Statistical Package for the Social Sciences for Windows version 12.0 (SPSS, Chicago, IL, USA). Continuous variables are reported as means ± standard deviations. Statistical significance was assessed using the Student's *t*-test and the paired *t*-test for continuous variables, and using the χ^2 ^test for categorical variables. A *P *value of < 0.05 was considered significant for the statistical tests.

## Results

### Epidemiological features of the patients with 2009 H1N1 virus infection

**Figure 1:**
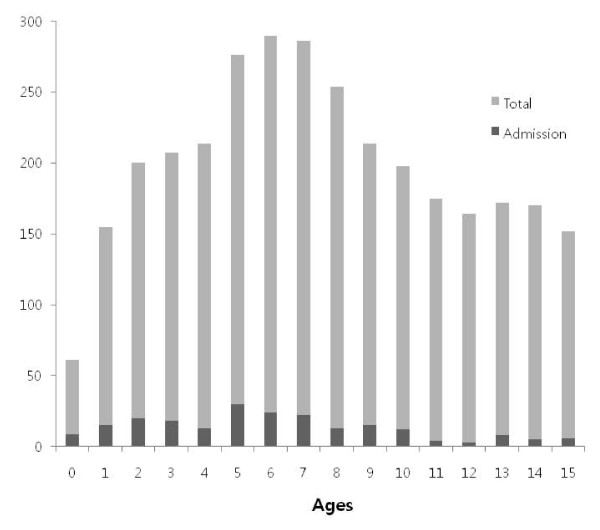
**Age distribution of the H1N1 virus infected patients**.

**Figure 2:**
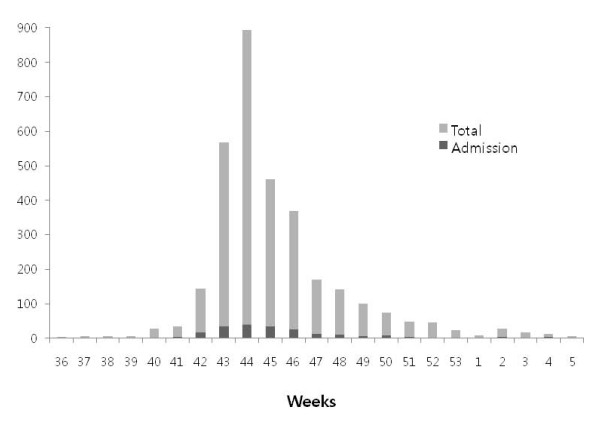
**Weekly frequencies of H1N1 virus infected patients**.

During the study period, 5,975 children (aged 2 months-15 years) with influenza-like illness were seen at our hospital. Among them, the 2,971 patients were positive by RT-PCR, and 217 patients were admitted to the isolation wards. The hospitalization rate was 7.3%. The mean age of the patients (2,971 cases) was 7.6 ± 4.1 years of age and the male-to-female ratio was 1.1:1 (1,581/1,390). The age distribution of the patients is shown in Figure 1. Children of all age groups except infants had a relatively even rate of infection (Figure 1, gray bars). The numbers of new patients each week is shown in Figure 2. There was an explosive pattern, with over three quarters of the cases occurring during a single month (the 43rd-47th weeks of 2009; 18 October to 14 November). The age distribution and the weekly case rate of the patients admitted to hospital (217 cases) are also shown on Figure 1 and 2 (black bars). These demonstrated similar patterns to those of the total patient cohort. For hospitalization rates, younger children (0-4 years, 9.8%, 75/762) showed higher admission rates than older children (5-9 years, 8.6%, 104/1216 and 10-15 years, 3.8%, 38/993).

### Clinical features of the patients admitted with 2009 H1N1 virus infection

In the 217 hospitalized children, the mean age was 6.2 ± 3.7 years, and the male-to-female ratio was 1.6:1 (132/85). Almost all the patients had a high fever (97.2%) and cough (92.6%) suggestive of severe infection such as pneumonia at the time of admission. Most of the patients were previously healthy although some had underlying diseases and only 17 patients had underlying disorders (7.8%), including 11 chronic pulmonary diseases (10 patients with bronchial asthma and one with past history of brochopulmonary dysplasia), 4 neuromuscular disorders (two with epilepsy and two with cerebral palsy), one with chronic liver disease, and one with nephrotic syndrome. The outcomes for these patients were uneventful, except for two asthmatic patients who developed a mild pneumonia. All inpatients received oseltamivir of the recommended doses for body weight and a broad-spectrum antibiotic (ampicillin/sulbactam). Two hundred and five patients (94.5%) received oseltamivir within 48 h of fever onset. The mean duration of fever before admission (including the day of admission) was 2.0 ± 0.8 d and 167 patients (77%) defervesced on the next day. Only seven patients had fever that persisted > 48 h after oseltamivir treatment. During hospitalization, five patients showed progressive pneumonia despite early antiviral therapy.

According to the initial chest radiographs, 80 patients had pneumonia and were divided into two groups: the bronchopneumonia group (49 patients) and the segmental/lobar pneumonia group (31 patients). In pneumonia patients, pneumonic infiltrations appeared within 48 h of fever onset in 68 patients (85%). When we analyzed the inpatients according to age (0-4 years, 75 patients; 5-9 years, 104 patients; and 10-15 years, 38 patients), the rates of pneumonia in each age group were 36.0% (27/75), 45.2% (47/104) and 8.8% (6/38), respectively. The severe pneumonia (segmental/lobar type) was predominant in the older age groups (14.8% (4/27), 48.9% (23/47) and 50% (3/6), respectively).

**Figure 3:**
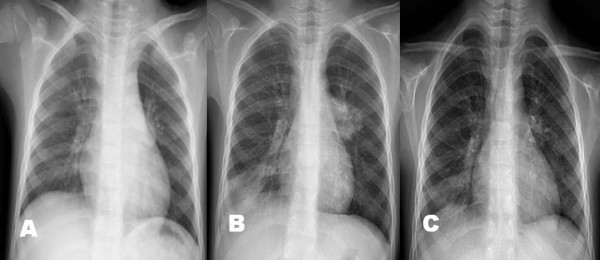
**The chest radiographs of a patients who had progressive pneumonia during hospitalization (male, 6-year old); on admission (Figure 3-A), before methyprednisolone (MP) treatment (2nd hospital day, Figure 3-B), and 1 day after MP treatment (Figure 3-C)**.

**Figure 4:**
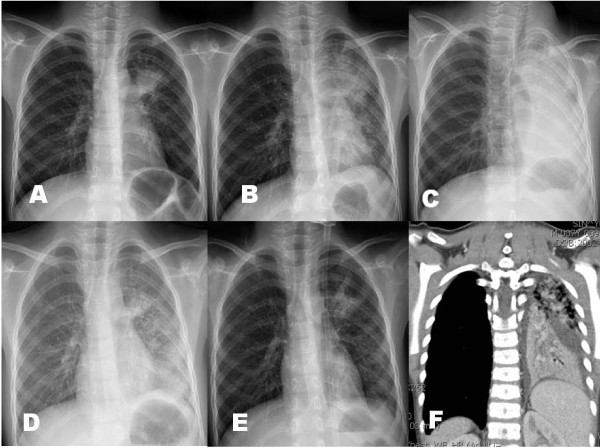
**The chest radiographs of another patient who had rapidly progressive pneumonia during hospitalization (male, 7-year old); on admission (Figure 4-A), 2nd hospital day a.m. (Figure 4-B), 2nd hospital day p.m. (Figure 4-C), 3 h after MP treatment (Figure 4-D), 15 h after MP treatment (3rd hospital day, Figure 4-E), and a chest computed tomography finding on 2nd hospital day p.m. (Figure 4-F)**.

**Figure 5:**
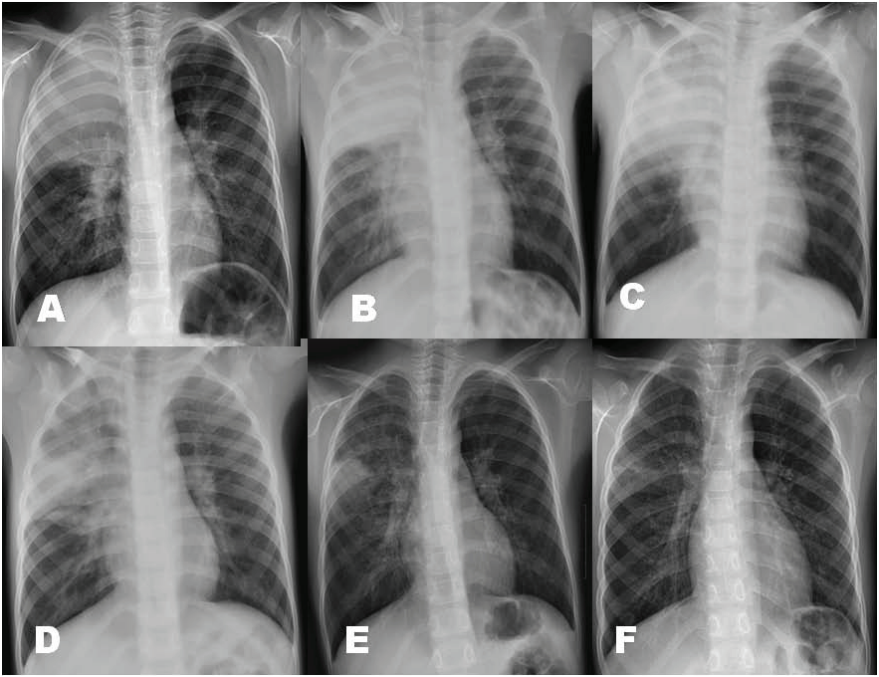
**The chest radiographs of a patient who were treated with MP and intravenous immunoglobulin (IVIG) (male, 7-year old); on admission (Figure 5-A), before MP treatment (Figure 5-B), after 2 doses of MP infusion (Figure 5-C), just after termination of IVIG infusion (Figure 5-D), 8 h after IVIG infusion (Figure 5-E), and 3 days after MP and IVIG treatment (Figure 5-F)**.

Among the 80 pneumonia patients, 17 patients showed severe respiratory distress with hypoxemia at presentation (12 cases) and during their hospital stay (5 cases). Arterial blood gas analysis was done in 15 patients, and 12 patients showed hypoxemia (PO_2 _< 60 mmHg in the room air). These patients received additional corticosteroids as soon as possible when indicated; 12 patients received intravenous MP (10 mg/kg/day, divided two doses, at presentation, 5 mg/kg/day at next day and then tapered off within a week) and 5 patients received oral prednisolone (1 mg/kg/day, divided 3 doses, for 3 days tapered off within a week). Six patients received early MP with osetalmivir which is recommended early use, before the positive RT-PCR results. Interestingly, all patients except one (age 4 years) were among the 5-9 years age group, and male patients were predominant (13/17). We performed serial chest radiographs of some cases in these patients. A 6-year-old male patient complained of one day fever and cough, and his initial chest radiograph showed few pulmonary infiltrations. On the following morning, he complained of severe dyspnea and showed chest radiographic infiltrations on right lower lung and left hilar regions. He had received two doses of oseltamivir before the MP treatment (Figure 3A-C). A 7 year-old male patient was presented with a day fever and severe cough and had a rapidly progressive pneumonia in which initial patchy infiltrates on left upper lobe progressed to total left lung consolidation within 12 h after admission. He was given three doses of oseltamivir before the MP treatment (Figure 4A-G). These two patients showed dramatic improvements in their clinical symptoms and radiographic findings within 24 h after MP treatment. A 7-year-old male patient with lobar pneumonia was treated with oseltamivir, MP and intravenous immunoglobulin (IVIG) treatment. He was admitted with fever, cough and progressive dyspnea of 2 days, and after MP (10 mg/kg/day, divided 2 doses) infusion, he showed persistent dyspnea and slightly aggravated pneumonic consolidation (Figure 5C). On the following morning, high-dose IVIG (1 g/kg) was infused for 6 hours. At the time of termination of IVIG, his clinical symptoms disappeared and a dramatic improvement of radiographic findings was observed within 6 hours after IVIG termination (Figure 5A-F). There was no adverse or rebound reaction in any patient treated with corticosteroids. The clinical symptoms of all patients improved within a day and their pneumonic infiltrations, regardless of severity, ceased immediately after corticosteroid treatment and disappeared within several days without adverse reactions.

Extrapulmonary manifestations (complications) of H1N1 infection were observed as four cases of febrile seizure, two cases of urticaria, one case of hepatitis and one case of myositis, and no cases of encephalopathy.

### Laboratory findings of the inpatients with 2009 H1N1 virus infection

When we compared the patients with and without pneumonia, and the patients with segmental/lobar pneumonia versus those with bronchopneumonia, there were significant differences in certain parameters between the groups. Compared with the group without pneumonia, the males were over-represented in the pneumonia group (*P *= 0.001) and longer hospitalizations (*P *< 0.001), higher values for hemoglobin (*P *= 0.03), WBC (*P *< 0.001) and CRP (*P *< 0.001) and lower values for lymphocyte differential (*P *< 0.001) (Table 1). The patients with the more severe type of pneumonia (segmental/lobar pneumonia) showed a higher mean age (*P *= 0.001) and leukocyte count (*P *= 0.01) and lower lymphocyte differential values (*P *< 0.001) compared with the group with bronchopneumonia (Table 2). In addition, the severe pneumonia patients who received corticosteroids (17 cases) had the highest leukocyte counts and CRP levels, and the lowest lymphocyte differentials (11 800 ± 3600/mm^3^, 3.0 ± 3.1 mg/dL, and 8.8 ± 6.3%, respectively) [10].

**Table 1:** Clinical and laboratory findings of patients infected with the 2009 H1N1 virus, with and without pneumonia

Group	No Pneumonia (n = 137)	Pneumonia (n = 80)	*P*
Clinical characteristics			
Mean age (y)	6.5 ± 4.0	5.5 ± 3.1	0.04
Male/Female	72/65	60/20	0.001
Duration of fever (d)			
Before admission	1.9 ± 0.9	2.0 ± 0.9	NS
Total	2.2 ± 1.0	2.6 ± 0.9	0.006
Hospitalization (d)	4.5 ± 1.4	5.6 ± 1.3	< 0.001
Oseltamivir, < 48 h (n,%)^a^	130 (95)	75 (94)	NS
Laboratory findings			
Haemoglobin (g/dL)	12.5 ± 0.9	12.8 ± 1.0	0.03
Leukocyte (× 10^9^/L)	6.5 ± 3.3	8.8 ± 3.7	< 0.001
Neutrophil (%)	59 ± 19	69 ± 20	< 0.001
Lymphocyte (%)	30 ± 17	21 ± 17	< 0.001
Monocyte (%)	10 ± 4	8 ± 4	0.005
Platelet (× 10^9^/L)	224 ± 58	241 ± 61	0.04
CRP (mg/dL)	1.2 ± 1.5	2.4 ± 2.5	< 0.001
ESR (mm/h)	16 ± 12	18 ± 13	NS

CRP; C-reactive protein, ESR; erythrocyte sedimentation rate. NS, statistically non-specific

^a ^Number (%) of patients who received oseltamivir treatment within 48 h of fever onset.

**Table 2:** Clinical and laboratory findings according to the severity of pulmonary lesions

Group	Bronchopneumonia (n = 49)	Segmental/Lobar (n = 31)	*P*
Clinical characteristics			
Mean age (y)	4.7 ± 2.9	6.9 ± 2.9	0.001
Male/Female	37/12	23/8	NS
Duration of fever (d)	2.6 ± 1.0	2.5 ± 1.0	NS
Hospitalization (d)	5.2 ± 1.2	6.2 ± 1.3	0.001
Oseltamivir, < 48 h (n,%)	46 (94)	29 (94)	NS
Laboratory findings			
Haemoglobin (g/dL)	12.6 ± 1.0	13.2 ± 0.8	0.006
Leukocyte (× 10^9^/L)	8.2 ± 3.5	9.7 ± 3.8	0.01
Neutrophil (%)	64 ± 21	77 ± 13	0.001
Lymphocyte (%)	26 ± 18	14 ± 11	< 0.001
Monocyte (%)	8 ± 3	7 ± 4	NS
Platelet (× 10^9^/L)	231 ± 58	255 ± 64	NS
CRP (mg/dL)	2.0 ± 2.1	3.0 ± 2.8	NS
ESR (mm/h)	18 ± 13	16 ± 13	NS

## Discussion

In this study, we found that children of all ages except infants had a relatively even rate of infection with the 2009 H1N1 influenza virus. This is in agreement with other studies showing that few children and young adults have immunity against a new viral infection [2,11,12]. Our study of all 2009 H1N1 virus-infected children, in a single hospital, throughout the epidemic may have epidemiological implications, along with other studies based on data from admitted patients or gathered during a portion of the pandemic period [11-14]. It has been reported that younger children (< 4-5 years old) with H1N1 virus infection tended to be admitted to hospital more frequently than older children or adults, and our results are compatible with these studies [2,3,6]. It has also been reported that certain risk factors are related to the likelihood of developing severe illness, including a younger age and particular underlying diseases, including obesity [6,7,15,16]. In this study, we did not analyze obesity and young age as risk factors, and only 17 patients (7.8%) had underlying diseases. Although the hospitalization rate was higher among the younger children (0-4 years of age), in this study, pneumonia and severe pneumonia were more prevalent in the 5-9-year-old group than in younger children.

The explosive increase in the infection rate during two months (mid-October to mid-December) of the study period may be a typical pattern of spread of an acute respiratory viral infectious disease that has a short incubation period (1-3 days). This pattern is similar to those reported in other regions of Korea and other countries [3,4,17]. Vaccination against the 2009 H1N1 influenza virus in Korea started on 18 November, 2009 for school-aged children, on 7 December, 2009 for children aged 6 months to 5 years and on 21 December, 2009 for adults with risk factors, which was after the peak of the epidemic. The rapid disappearance of the spread of infection might be the effect of vaccination and the strengthened individual hygiene, but the epidemiologic pattern suggests that an unknown herd immunity against a new viral infection might also be responsible [3,17].

It is reported that patients with 2009 H1N1 virus infections had a relative leukopenia with lymphopenia [18,19]. However addition to this finding, we found in the present study that the patients with pneumonia had a higher WBC count with lower lymphocyte differential, and the more severely affected patients had the highest WBC with the lowest lymphocyte differential in the early stages of the infection (within two days of fever onset). Previous human studies of H5N1 virus infections have revealed that a lower lymphocyte count was associated with a poor outcome [20], and mice infected with influenza viruses showed a lymphopenia and the H5N1 subtype was associated with marked lymphopenia with total lymphoid depletion [21]. Therefore peripheral lymphocyte may be associated with the pathogenesis of acute lung injury in influenza virus infections [9]. Altered CRP and ESR values were not prominent in 2009 H1N1 virus infections, but higher CRP values were associated with a more severe illness.

The male-to-female ratio was 1.1:1 among all patients, and 1.6:1 among the admitted patients. Furthermore, male patients were predominant among those with pneumonia (3:1, 60/80) and those with respiratory distress who received corticosteroids (3.3:1, 13/17). Other epidemiologic studies on children have reported a male predominance [16], but some studies on inpatients have reported a female predominance [4,7]. These findings suggest that genetic factors and possibly environmental factors are associated with the pathogenesis of 2009 H1N1 virus-associated pneumonia.

Regarding the pathogenesis of lung injury in influenza virus infections, it has been believed that the viruses from upper respiratory tract spread to lower lung tissues and elicit the cytopathic reaction. However, some clinical and experimental studies have suggested that the innate and/or cell-mediated immune reaction (T cell) with excessive production of cytokines of the host may also be responsible for the lung injury in influenza virus infection [9,22-25]. We experienced no intensive care patient in this series despite large subjects of the study, and it may be, at least in part, explained by a rapid use of corticosteroids on the patients with severe pneumonia [9,10]. Because this pandemic occurred over 40 years after last pandemic (Hong Kong flu), there have been no controlled-clinical trials for the efficacy of corticosteroids on influenza virus infections, although yearly seasonal influenza and small cases of sporadic H5N1 avian influenza virus infection have occurred during inter-pandemic period. The beneficial effect of corticosteroids in pneumonia caused by influenza virus infections may have resulted from reduction of systemic inflammation caused by immune cells and cytokines [26,27]. Our treatment policy which needs to be proven by controlled clinical studies in coming pandemics or in other influenza virus infections, may help to reduce the morbidity and possibly prevent the progression to fatal pneumonia [9,10]. We have reviewed the rationale of our corticosteroid treatment and the host immune responses to viral insults in influenza virus infections, and proposed a new concept for the pathogenesis of acute lung injury in influenza virus infections, using a 'protein homeostasis system' of the host [9].

It has been reported that antiviral therapy (oseltamivir) is effective in the acute stages of influenza infection, including H1N1 virus infection in humans and experimental animals [4,28]. In agreement with this, we found that the majority of patients (97%) defervesced within 48 h after medication and most pneumonic infiltrations in pneumonia patients had improved at discharge.

There are some limitations to this study. Compared with other studies, we had many uncomplicated inpatients owing to flexibility of our admission policy. Because we did not perform extensive microbiological testing, such as viral cultures, paired-sample serologic studies and PCR for other pathogens, we cannot rule out the possibility of co-infection with other respiratory pathogens.

## Conclusions

In pandemic 2009 H1N1 virus infections, children of all ages were evenly affected, and males were predominant in pneumonia patients. Early antiviral treatment was very effective in inducing rapid defervescence for the patients. The patients with 2009 H1N1 infections showed lymphopenia, and its severity was associated with the severity of the illness in the early stages. Together this finding, the rapid improvement in clinical signs and the prompt resolution of severe pneumonic consolidations after immune-modulator (corticosteroid and IVIG) treatment suggest that the mechanisms of lung injury in this infection may be associated with the cell-mediated immune response of the host, rather than virus-induced cytopathies.

## Completing interests

The authors declare that they have no competing interests.

## Authors' contributions

All authors read and approved the final manuscript. KYL had primary responsibility for concept, design of the study and writing the manuscript; JWR participated in the preliminary data collection, data analysis and writing the manuscript; YSY participated in patient care and data analysis; JHK contributed to the interpretation of the data and editing of the manuscript and supervised the design and execution of the study; JCK participated in reading of chest radiograph of the patients

## Pre-publication history

The pre-publication history for this paper can be accessed here:

http://www.biomedcentral.com/1471-2334/11/225/prepub
